# At-Sea Measurement of the Effect of Ship Noise on Mussel Behaviour

**DOI:** 10.3390/s25133914

**Published:** 2025-06-23

**Authors:** Soledad Torres-Guijarro, David Santos-Domínguez, Jose M. F. Babarro, Laura García Peteiro, Miguel Gilcoto

**Affiliations:** 1 atlanTTic Research Center, Universidade de Vigo, 36310 Vigo, Spain; dsantos@gts.uvigo.es; 2 Instituto de Investigaciones Marinas de Vigo, IIM-CSIC, 36208 Vigo, Spain; jbabarro@iim.csic.es (J.M.F.B.); lpeteiro@iim.csic.es (L.G.P.); mgilcoto@iim.csic.es (M.G.)

**Keywords:** *Mytilus galloprovincialis*, mussel, underwater noise, particle motion, behaviour

## Abstract

Anthropogenic underwater noise is an increasing form of pollution that negatively affects biota. The effect of this pollutant on many marine species is still largely unknown, especially those that are more sensitive to particle motion than to sound pressure. In these cases, experiments at sea are necessary, due to the difficulty of recreating the particle movement of a real acoustic field under laboratory conditions. This work aims to contribute to the knowledge of the effect of ship noise on the behaviour of mussels (*Mytilus galloprovincialis*), performing measurements at sea on a real mussel cultivation raft for the first time. The study is carried out on cluster-forming individuals living in the rafts where they are cultivated. Their behaviour is monitored by means of valvometry systems, which measure the magnitude of shell opening using a High-Frequency Non-Invasive (HFNI) system. Simultaneously, the acoustic field generated by the abundant traffic in the area is measured. The results show cause-and-effect relationships between ship noise and valve closure events.

## 1. Introduction

Underwater noise of anthropogenic origin is an increasing form of pollution that negatively affects biota. The effect of this pollutant on many marine species remains largely unknown and depends on both the characteristics of the sound (level, frequency, power distribution, distance to the source, duration, etc.) and the sensitivity of the species under study.

Although the effects of underwater noise on marine fauna are complex and not yet fully understood, its potential for harm is well recognised, and policy measures are already being developed. The Marine Strategy Framework Directive (MSFD) includes a specific descriptor—Descriptor 11—focused on underwater noise as part of its criteria for achieving ‘Good Environmental Status’ (GES). These descriptors aim to assess the overall health of the marine environment, encompassing both ecological aspects, such as biodiversity and food webs, and human-induced pressures, including commercial fisheries, marine litter, chemical contaminants, and the input of energy, such as underwater noise [1,2].

Over the past decades, significant progress has been made in understanding the effects of noise, particularly in relation to behavioural responses in marine mammals [3,4]. This advancement has been driven by innovative technologies, large-scale coordinated field studies, and dedicated funding. However, our knowledge of how noise impacts fish, and especially invertebrates, remains limited and continues to lag behind [5].

Marine invertebrates represent the largest proportion of marine biomass, are indicators of environmental health, and play a key role in marine ecosystems [6]. They perform essential ecosystem services, such as bioturbation (e.g., infaunal bivalves buried in the sediment), the removal of a large part of the nutrients from the water column through the filtration of large volumes of water (bioextraction) that could otherwise generate processes of eutrophication and/or anoxia, the stabilisation of the coastline against the impact of waves and coastal erosion processes, aquaculture as a source of high nutritional quality protein, and low-carbon footprint production systems representing socio-economic sectors of great relevance. In addition, many of these organisms are sessile or have relatively little capacity for movement, which is why they are used as environmental sentinels (biosensors) to report the magnitude and effects of abiotic and/or biological environmental changes.

In recent decades, numerous studies have tried to assess in different ways the effects of different stressors on bivalves [7,8]. But only recently have experiments been conducted in relation to the effect of noise as a stressor, and how anthropogenic or low frequency sounds can interfere with the behaviour, biochemistry, and ecophysiology of bivalve species such as *Mytilus edulis* [9,10,11,12,13], *Mytilus galloprovincialis* [10,14,15], *Sinonovacula constricta* [16], *Perna perna* [17], *Crassostrea virginica* [18] or *Pinctada radiata* [19], among others.

Valve gaping behavioural impairments related to underwater noise are the most frequently reported effects on bivalves [9,10,11,12,13,14,15,16,17,18,19]. Bivalve gaping behaviour can be monitored remotely and continuously in natural environments over extended periods, using HFNI (High-Frequency Non-Invasive) valvometers, allowing for the use of bivalves as biosensors to detect and provide early-warning signals of changes in GES [20]. Specifically, this technique could be used to monitor specific indicators for underwater noise, with potential applications at harbours or aquaculture sites. Thresholds and restrictions should be set according to different species, environment, and equipment before establishing valvometry as a standard monitoring technique for detecting noise-induced stress in bivalves.

All the dose–response studies carried out to date have been conducted in a laboratory setting. In these experiments, specimens are maintained within a water tank in controlled conditions, with the use of a loudspeaker to reproduce acoustic stimuli and, on certain occasions, a sensor or camera to analyse their behaviour. This experimental approach has intrinsic limitations related to the acoustic field that can be generated under these conditions and how it is perceived by the animals under study, making it more challenging to interpret the results obtained as well as to extrapolate them to environmental conditions in nature. In a sound wave, particles in the medium (e.g., water molecules and suspended particulate matter) oscillate about their resting position (i.e., particle motion), causing local compressions and expansions (i.e., sound pressure) [21]. All sound causes fluctuations in both particle motion and sound pressure. Sound pressure is measured using hydrophones, while particle motion, which can be described by displacement, velocity, or acceleration, can be measured by accelerometers, geophones, or hydrophone pairs, or calculated (except for directional information) from sound pressure measurements under certain conditions detailed below.

Invertebrates lack an ear, and therefore do not detect sound through sound pressure, but they do possess statocysts that sense particle motion, allowing them to maintain equilibrium in the water column and to perceive gravity [22]. In addition, in bivalve mollusc species such as scallops, the abdominal sensory organ (ASO) has been shown to be very sensitive to water-borne mechanical vibrations [23] and thus to particle motion.

Therefore, experiments on the effect of noise on invertebrates should be carried out with the particle motion as the acoustic variable of reference, i.e., the acoustic field to which individuals are subjected should be generated in such a way that the particle motion can be controlled. The main source of anthropogenic noise in bivalve culture areas usually comes from maritime traffic (goods and passenger transport, sea farming, fishing, etc.). For example, in the Ría de Vigo, an area of high aquaculture production in NW Spain, it has been shown that the main noise stressor in some of the mussel raft parks where *Mytilus galloprovincialis* is cultivated is the noise of passenger ships that continuously cross the ria [24]. The difficulty in studying the effect of ship noise is due to the lack of the necessary equipment to do so in a laboratory: a loudspeaker submerged in a small tank playing a recording of boat noise is unable to generate a particle motion similar to that actually caused by a ship in the coastal zone [25]. The scientific community is aware of this limitation, and there is at least one prototype in development of a laboratory tank for invertebrates where particle velocity can be controlled [26]. But until such equipment is fully tested and widely available, experiments at sea are essential, where the sound field that actually affects the species, with its associated particle motion, is already present.

For this reason, the study presented here has been carried out at sea, and to our knowledge, it is the first time that the effect of anthropogenic noise on the behaviour of mussels in their cultivation arrangement in rafts has been studied. The species studied is *Mytilus galloprovincialis*, during its cultivation on floating platforms (rafts) in the Ría de Arousa (Spain). Its behaviour is monitored by measuring the valve opening rhythms in real time, non-invasively, and at high frequency. Given that mussels in culture are open practically all the time [27], valve closure either as persistent closure or as micro-closures (high frequency micro-movements of the valves, not detectable to the human eye) respond to a certain degree of environmental stress, which has been observed in the presence of toxic phytoplankton in other studies [28]. In our case, the stressor under study is particle motion produced by the acoustic field and is estimated from in situ pressure measurements. Its correlation with valve gaping allows us to analyse the effect of ship traffic noise on individuals of this species and to consider the potential use of *M. galloprovincialis* as a biosensor for this environmental stressor.

## 2. Materials and Methods

### 2.1. Experimental Sites

**Figure 1:**
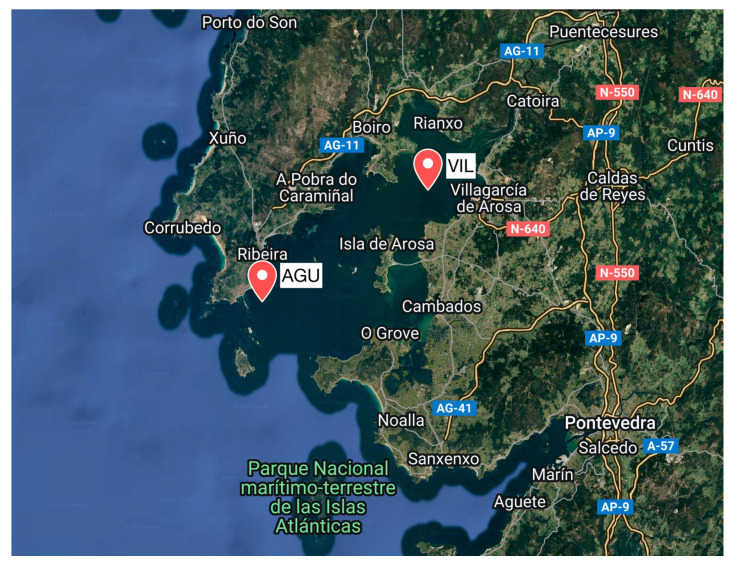
Location of the rafts with the mussels studied, in the Ría de Arousa (NW Spain).

The experiment was carried out on two mussel rafts between 6 September and 1 November 2023, following the standard cultivation practices commonly used in the region for *Mytilus galloprovincialis*. The rafts were located in the Ría de Arousa (Figure 1; Table 1), a flooded valley on the northwest coast of Spain (the world’s third-largest mussel producer), which hosts the majority of the country’s mussel production, with over 3300 cultivation rafts and significant maritime traffic in the area.

The two rafts chosen for the study have very different habitats [29]: the VIL raft is in sheltered, shallow waters, less salty due to the proximity to the mouth of the river Ulla and with low ambient noise; the AGU raft is in more exposed, rougher and deeper waters, with higher ambient noise from wind and waves. At both rafts, mussel behaviour, jointly with acoustic field measurements, was continuously monitored.

### 2.2. Mussel Behaviour Monitoring

**Figure 2:**
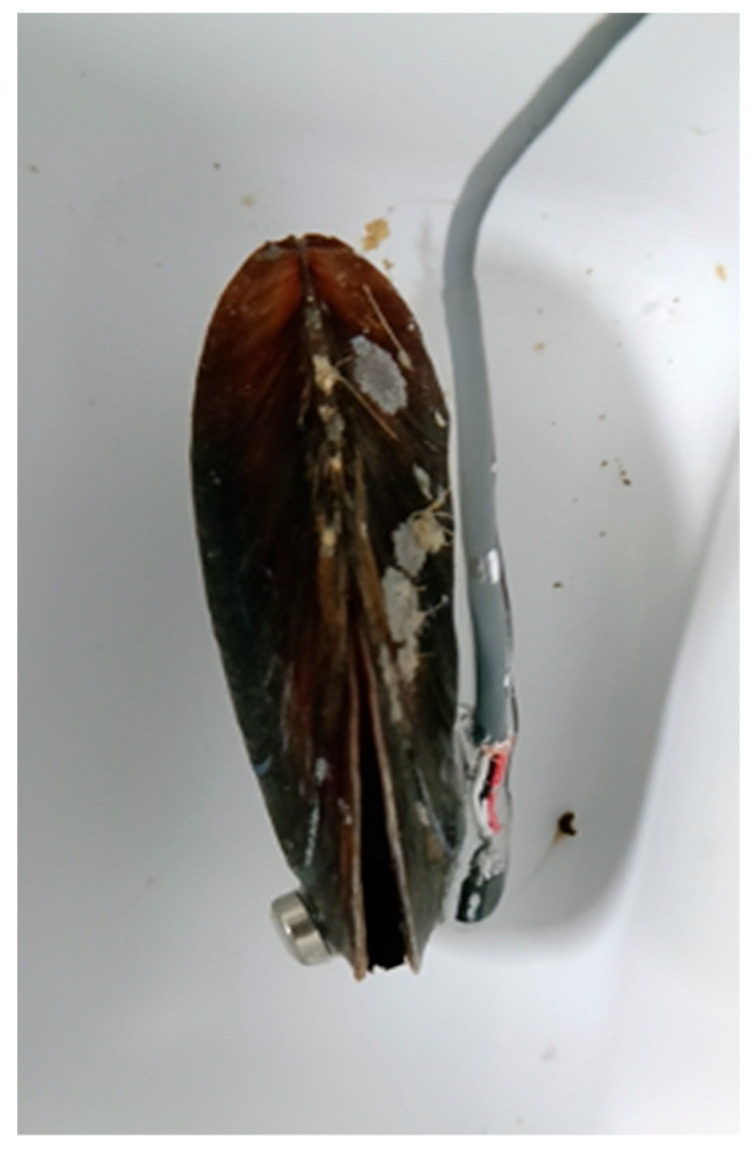
Valvometry sensor attached to the shell of a mussel in a lab setup: magnet (left valve) and Hall effect sensor (right valve).

The shell opening of each mussel was measured with a valvometry system [30] consisting of a Hall effect sensor (model 49E) and a small magnet (4.8 mm diameter × 0.8 mm high) attached with cyanoacrylate to each of the two valves, as shown in Figure 2. These two elements are placed at the furthest point from the umbo, where the opening is maximum. Both elements have negligible (non-invasive) weight for the individual: 0.1 g for the magnet and 0.5 g for the Hall sensor.

The Hall sensor measures the magnetic flux density produced by the magnet, which is inversely proportional to the distance between the magnet and the sensor, so the more open the shell is, the smaller the signal generated by the sensor. This signal is acquired with a monitoring system based on an Arduino device. Hall sensors were attached to 26 mussels per raft (14 connected directly to the Arduino’s ADCs, and 12 more through three external 4-channel analogue-to-digital converters (ADCs)), allowing for monitoring gapping behaviour of 52 mussels in total at a sampling rate of 10 Hz. Each monitoring system also collects information on water temperature, the rope weight with a load cell sensor, and the raft movement with an inertial measurement unit (IMU). A real-time clock (RTC) module timestamps each data record, which is stored on a microSD card. The information is sent every 20 min to our servers onshore via mobile telephony, and the local copy on the memory card is downloaded on monthly sampling visits. The monitoring systems are powered by batteries recharged by solar panels.

**Figure 3:**
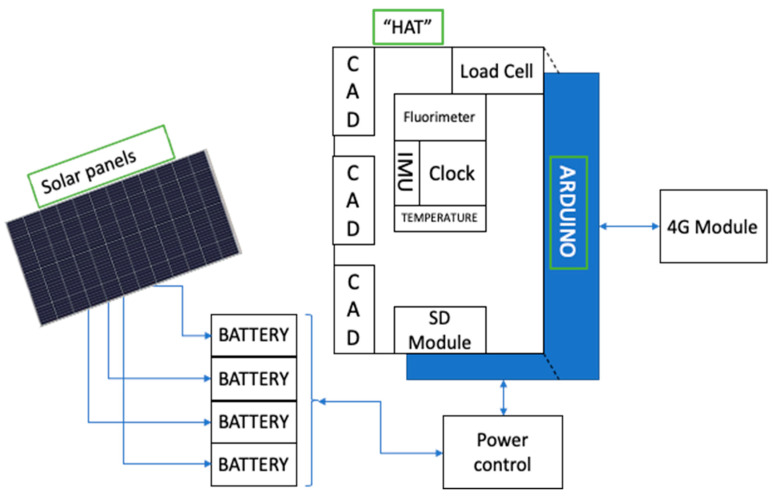
Schematic of the raft monitoring system.

Figure 3 shows a schematic of the complete monitoring system. A HAT (stackable add-on functionality module) including the 3 external ADCs, load cell, fluorometer, IMU sensor, water temperature sensor, clock module, and SD module is connected to the Arduino.

**Figure 4:**
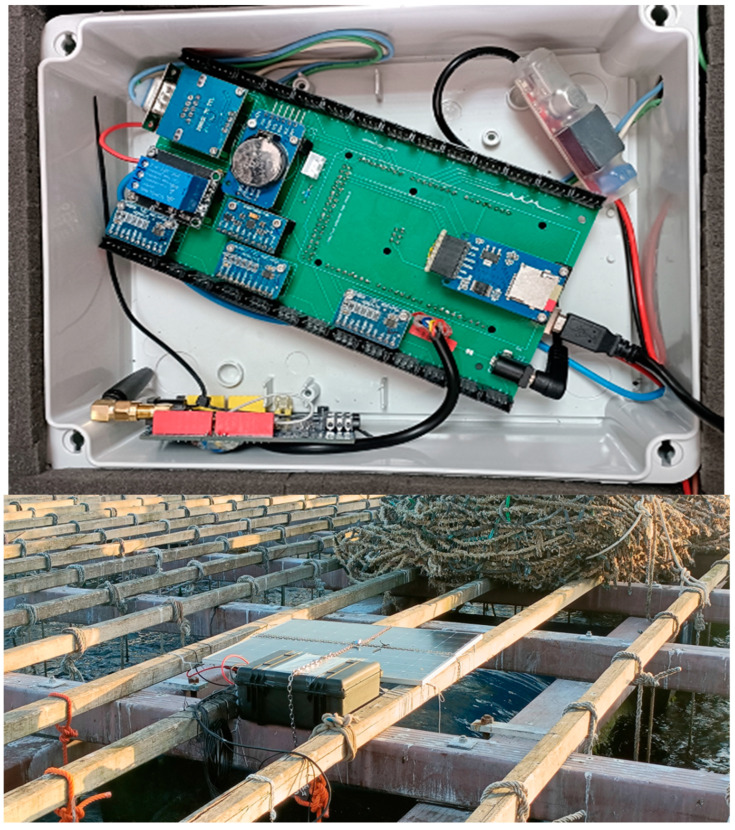
(**Top**): electronics; (**Bottom**): the Peli case with the electronics and the solar panels installed on the raft.

**Figure 5:**
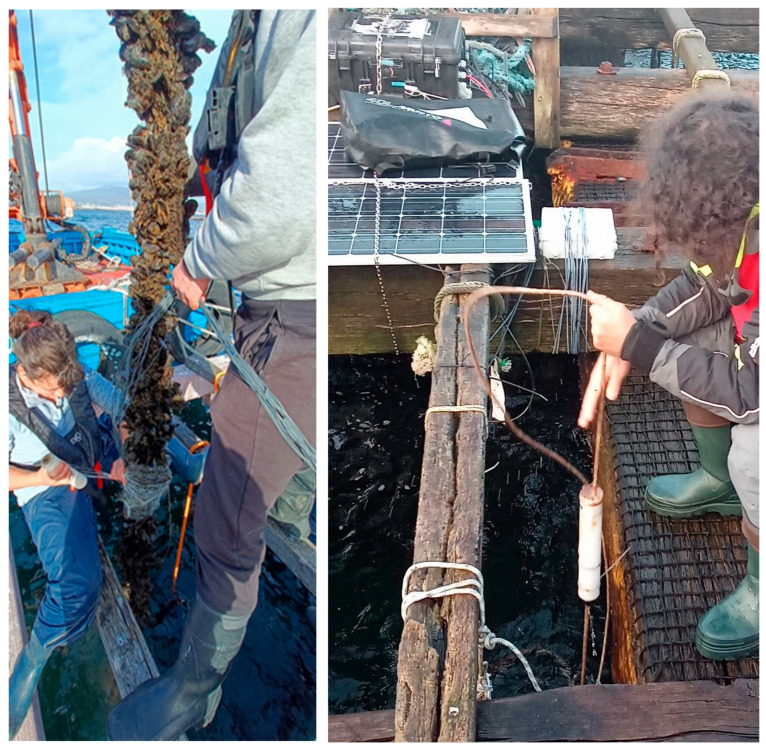
(**Left**): Operation of attaching the sensorised mussels to a rope of the raft; (**Right**): hydrophone deployment operation, the monitoring system integrated in the Peli case with the electronics can be seen in the upper left corner, behind the solar panels.

Figure 4 and Figure 5 show photographs of the electronics, the Peli case containing it, and the solar panels installed on the raft, the operation of attaching the sensorised mussels to a rope of the raft, and a hydrophone mooring operation where the monitoring system installed in one of the rafts can be seen.

Curation of the valvometry data is performed by visually analysing the signals and comparing them with those previously obtained in laboratory experiments [15,28], in which the good condition of the cables, sensors, and magnets could be visually verified. This procedure discards signals whose value ranges are out of the usual magnitude, or show anomalous behaviour, caused by a bad state of magnet or sensor fastening, the cutting of the wires, or the detachment of the mussel clusters from the rope.

**Figure 6:**
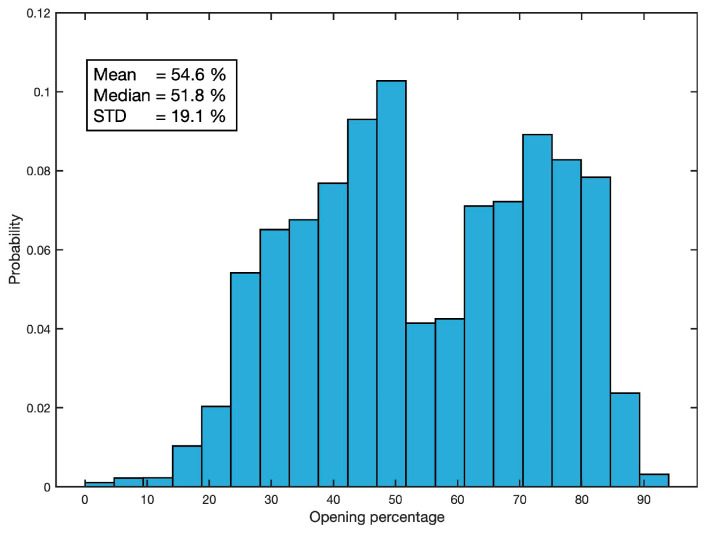
Statistical description of the valvar aperture.

The cured data are processed to convert the magnetic field values, provided by the sensors, into percentages of valve opening. This is performed for each mussel individually by assigning the maximum magnetic field value (interpreted to be the 0% valve opening value), and the minimum (assumed to be 100% opening). Finally, a 10-sample median filter is applied to reduce the sampling frequency to 1 Hz, and a 30-sample moving average smoothing is applied to reduce high-frequency valve closing unrelated to acoustic noise-induced mussel behaviour. Figure 6 shows the statistical description of the valvar aperture resulting from this processing in terms of its histogram, mean value, median, and standard deviation.

### 2.3. Acoustic Field Measurement

Systems for measuring particle motion, which is the acoustic variable that mussels perceive (accelerometers, geophones, or hydrophone pairs), need to be installed in such a way that they move as little as possible, so that their own movement does not mask the movement of the water particles they are intended to measure. For this reason, they are usually installed on the seabed and are fitted with a calibrated suspension system that minimises the movement of the sensor in the water [25]. This deployment is incompatible with the measurement of the acoustic field affecting the mussels monitored in this study, because they hang from rafts at a considerable distance from the bottom. The chosen alternative is therefore to suspend a hydrophone, whose measurements are much more immune to raft movement, and estimate particle movement from its measurements.

Given the characteristics of the experimental site (floating platforms not connected to land), and in order not to overload the power supply and the data storage and transmission capacity of the monitoring systems installed in the rafts, it was decided to use autonomous hydrophones with batteries and local data storage (Marsensing digitaHyd SR-1). They are deployed by hanging from a line from each raft, at a depth of 6 m, corresponding to half the mussel ropes’ length. The line is suspended from a spring to minimise the hydrostatic noise produced by the vertical oscillations of the raft caused by waves, and is ballasted at its lower end. During the month-long experiment, 4 trips were made to the two rafts to install and clean the hydrophones, and to replace batteries and data storage cards.

The hydrophones were programmed to record the sound in 3 min files in wav format, with a gain of 1x, a resolution of 16 bits/sample, and a sampling frequency of 52,734 Hz. With this configuration, the effective frequency range, between 63 Hz and 20 kHz, covers the significant sound emission from ship traffic, the main source of anthropogenic noise in the area.

The calculation of the sound pressure level from the sound recordings is performed in 1 s segments, which are transformed to the frequency domain using the Fourier transform. The sensitivity curve of each hydrophone is then applied to obtain the spectral sound pressure density P(f) in dB re 1 μPa with a time resolution of 1 s and frequency resolution of 1 Hz.

The particle motion is described by the displacement, velocity, or acceleration of the water molecules due to the propagation of the sound wave. These three variables can be calculated, except for the direction information, from the measured sound pressure, under certain conditions. Thus, when the acoustic field can be properly approximated to a plane wave or spherical propagation from a monopole, then it is possible to calculate the magnitude of the particle velocity from a sound pressure measurement, using the following expression [25]:

(1) $$u\left(t\right)=\frac{p(t)}{\rho c_{w}},$$

where *u* is the magnitude of the particle velocity as a function of time, *p* is the sound pressure, *ρ* is the density of water, and $c_{w}$ is the speed of sound propagation in water.

The monopole condition (point source radiating equally in all directions) is difficult to fulfil in our case, since the sound sources are ships of several metres in length that can hardly be modelled as point sources. The plane wave hypothesis implies that the wave propagates far enough away from its source for the wavefront to be considered flat, and that the propagation occurs without interference from other waves, including reflections. The applicability of this hypothesis depends on the frequencies of interest and the properties of the waveguide. These properties are the water depth, the distance to the sound source, the type of source, and the sound velocities in the water and sediment. In practice, the plane wave approximation is reasonable when two conditions are met [25]:

(1) The sound source is at a distance greater than one wavelength at the lowest frequency of interest, and;
(2) The lowest frequency of interest is higher than the cut-off frequency of the waveguide formed by the bottom and the sea surface.

To specify when condition (1) is met, we start from the relationship between wavelength and frequency in a sound wave:

(2) $$\lambda =\frac{c_{w}}{f},$$

where *λ* is the wavelength and *f* the frequency.

The lowest frequency of interest in our experiment is 63 Hz, which is the lower cut-off frequency of the hydrophones used. At this frequency, the wavelength is approximately 24 m, which means that ships passing at a distance from the hydrophone greater than 24 m produce an acoustic field that meets condition (1). In practice, during our experiment, we applied this condition by discarding sound and valvometry records during ship passes at shorter distances, calculated from Automatic Identification System (AIS) data. In the case of the raft service vessels, which usually sail in their vicinity and do not normally carry AIS, we determined by in situ recordings that at distances greater than 24 m they generate levels below 140 dB, and we eliminated from our analysis the records with levels above this value.

To determine when condition (2) is met, we start from the expression of the waveguide cut-off frequency:

(3) $$f_{0}=\frac{c_{w}}{4D\sqrt{1-\left(\frac{c_{w}}{c_{b}}\right)}},$$

where *f*_0_ is the cut-off frequency, D the depth of the water channel, and $c_{b}$ the sound propagation velocity in the bottom sediment.

The bottom of the experimental area is a mixture of mud and sand, so a reasonable value for $c_{b}$ is 1600 m/s [31]. Taking the depth of the VIL raft, 20 m (see Table 1), which is in shallower water, is the most unfavourable, and with an average value of 1500 m/s for $c_{w}$, we obtain a waveguide cut-off frequency of 54 Hz, below the lower cut-off frequency of our equipment. Therefore, condition (2) is fulfilled for all frequencies of interest.

As a conclusion, it is feasible to calculate the particle motion from the sound pressure measurement made with a single hydrophone, under the plane wave hypothesis, for frequencies above 63 Hz, provided that the ship producing the measured noise is at a distance of more than 24 m from the hydrophone. On the other hand, it must be taken into account that surface and bottom reflections influence the degree to which it is reasonable to assume that the plane wave condition is fulfilled. For this reason, a hydrophone installation depth of 6 m has been chosen, neither too close to the surface nor too close to the bottom.

In practice, the estimation of the magnitude of the particle motion from the sound pressure has been made in the frequency domain using expression (4), equivalent to (1),

(4) $$U\left(f\right)=\frac{P(f)}{\rho c_{w}},$$

where $U\left(f\right)$ is the magnitude of the particle velocity spectral density and P(f) is the sound pressure spectral density.

Since displacement, velocity, and acceleration are related to each other by derivative in time, the properties of the Fourier transform allow us to express

(5) $$\Delta \left(f\right)=\frac{U(f)}{2\pi if},$$

(6) $$A\left(f\right)=2\pi ifU(f),$$

where Δ(f) and $A\left(f\right)$ represent the particle displacement and acceleration spectral densities, and $i=\sqrt{-1}$.

The particle displacement variables estimated using this methodology have, like the sound pressure spectra from which they are derived, a time resolution of 1 s, a frequency resolution of 1 Hz and a frequency range between 63 Hz and 20 kHz. The spectral densities of the four acoustic variables (sound pressure, acceleration, velocity, and displacement) can be integrated in a certain frequency band or in the complete frequency range to obtain their global value, in the time domain, with a sampling frequency of 1 Hz.

To estimate the robustness of the cause-and-effect relationship between particle motion and valve aperture, we employed cross-correlation. This function measures the resemblance between two variables for different time offsets between them, to account for possible lags between cause and effect. The cross-correlation of two jointly stationary random processes, $x_{n}$ and $y_{n}$, is given by

(7) $$R_{xy}\left(m\right)=E\left\{x_{n+m}y_{n}^{*}\right\}=E\left\{x_{n}y_{n-m}^{*}\right\},$$

where −∞<n<∞, the asterisk denotes complex conjugation (which can be ignored in our case when working with real variables), m is the sample offset, and E is the expected value operator. Since we have only a finite segment of a random process realisation, we will estimate the cross-correlation by the expression

(8) $${\hat{R}}_{xy}\left(m\right)=\left\{\begin{array}{cc} \overset{N-m-1}{\underset{n=0}{\sum }}x_{n+m}y_{n}^{*}, & m\geq 0,\\ {\hat{R}}_{yx}^{*}\left(-m\right), & m<0, \end{array}\right.$$

where N is the length of both variables. To obtain a normalised estimator, we subtract from each variable its mean value and normalise the result of the calculation so that the autocorrelation for displacement m=0 is unity. We thus obtain the cross-correlation coefficients $R_{xy,coeff}(m)$, which take values between +1 and −1,

(9) $${\hat{R}}_{xy,coeff}\left(m\right)=\frac{1}{\sqrt{{\hat{R}}_{xx}\left(0\right){\hat{R}}_{yy}\left(0\right)}}{\hat{R}}_{xy}\left(m\right).$$

The largest absolute value of the sequence $R_{xy,coeff}(m)$ is an estimate of the linear dependence (positive or negative) between *x* and *y* for any time shift between the two variables. In our case, we bound this shift to ±600 samples (10 min) to accommodate the reaction time of the mussels, and possible slippage between the clocks of the two signals. We therefore define the linear dependence between *x* and *y*, ${LD}_{xy}$, as

(10) $${LD}_{xy}=\max\left(\left|{\hat{R}}_{xy,coeff}\left(m\right)\right|\right),\;for-600<m<600.$$

## 3. Results

### 3.1. Description of the Acoustic Environment of the Experimental Sites

After the experimental phase, more than 50 h of sound pressure measurements, corresponding to non-contiguous intervals on different days and times within the experimental period, are available for each of the experimental sites.

**Figure 7:**
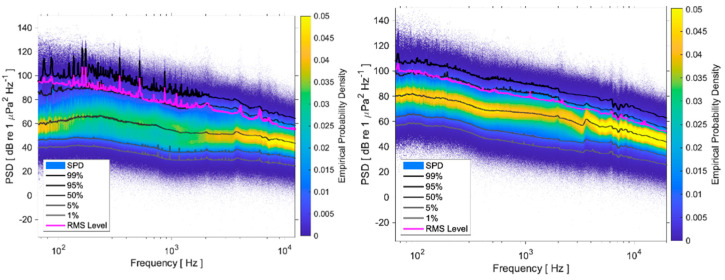
Sound power, Spectral Probability Density, and exceedance curves for 1, 5, 50, 95, 99% of the time in the two rafts: (**Left**): VIL; (**Right**): AGU.

Based on these measurements, the acoustic environment can be described by sound power Spectral Probability Density, and exceedance curves, which are shown in Figure 7 for both locations.

Comparison of graphs in Figure 7 shows that the noise environment at AGU is significantly louder than at VIL: the background noise level, described by the 1% curve, is about 20 dB higher at AGU than at VIL, while the 99% curve, associated with the noisiest events, is about 10 dB higher at AGU. These results are related to the higher wave noise at AGU, located very close to the northern mouth of the estuary. On the other hand, the VIL curves show tonal components of frequencies below 250 Hz, related to the engine and propeller noise caused by boat traffic, which is more intense and closer in VIL than in AGU.

**Figure 8:**
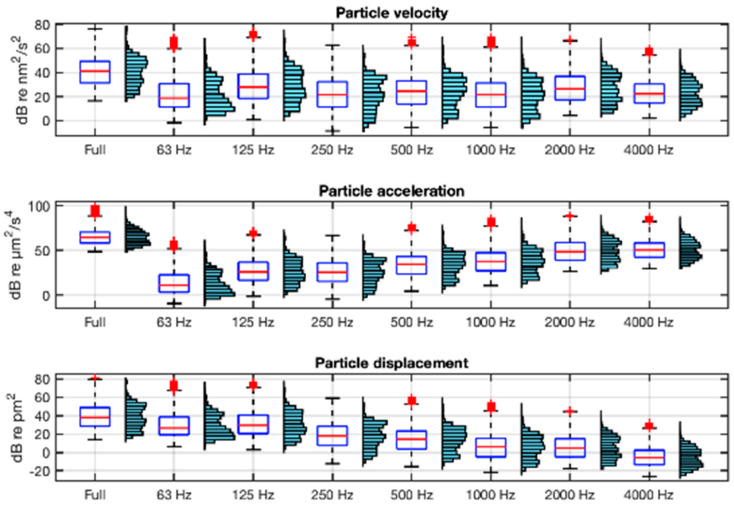
Boxplots and histograms of the full-band and octave-band for particle motion variables: velocity, acceleration, and displacement in VIL. The boxplot box is defined between the 25th and 75th percentiles, with whiskers up to 1.5 times the interquartile range; in red, the median and outliers.

**Figure 9:**
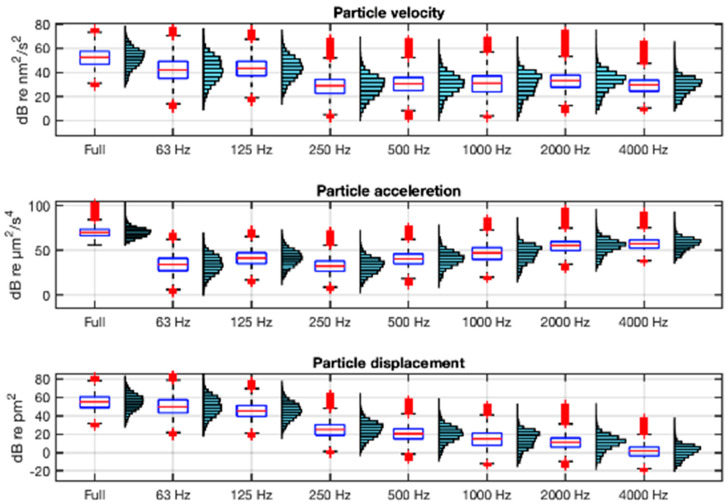
Boxplots and histograms of the full-band and octave-band particle motion variables: velocity, acceleration, and displacement in AGU. The boxplot box is defined between the 25th and 75th percentiles, with whiskers up to 1.5 times the interquartile range; in red, the median and outliers.

The description of both acoustic environments in terms of the particle motion variables is shown in Figure 8 and Figure 9 using the units recommended in [21]. They show, in boxplot and histogram format, the velocity, acceleration, and displacement levels in full band (63 Hz−20 kHz) and in octave bands (from 63 Hz to 4 kHz) integrations of their respective spectra. In the calculation of these statistics, ship passes within 24 m of the hydrophones have been excluded in order to maximise the accuracy of the estimation of particle motion from sound pressure.

As can be seen in Figure 8 and Figure 9, the frequency distribution of the velocity is parallel to that of the sound pressure (Figure 7), as indicated by Equation (4). In contrast, the acceleration has an increasing spectral tilt with frequency and the displacement a decreasing one, as indicated by Equations (5) and (6). The range of full-band acceleration values is 25–99 dB re µm^2^/s^4^ with a mean value of 68 dB re µm^2^/s^4^ at VIL, and 56–103 dB re µm^2^/s^4^ and a mean of 72 dB re µm^2^/s^4^ at AGU.

**Figure 10:**
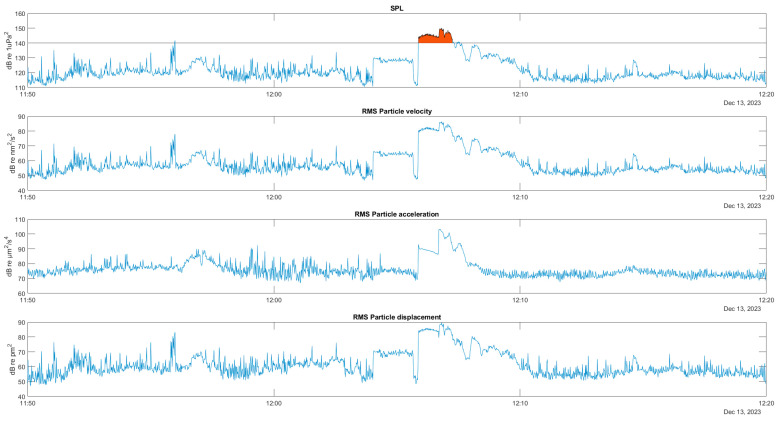
Time series of full band pressure and particle motion: velocity, acceleration, and displacement, for a close pass of a raft service vessel. In red SPL above 140 dB.

An example of the behaviour of the acoustic variables over time is shown below. The first graph in Figure 10 shows the time evolution of the full-band sound pressure in a segment of a recording containing the sound of the engine of a raft service vessel very close to the hydrophone installed at AGU. The area marked in red corresponds to a sound pressure level higher than 140 dB, which indicates that the distance to the hydrophone was probably less than the 24 m that guarantees the fulfillment of the plane wave hypothesis. The following graphs show the velocity, acceleration, and particle displacement estimated from this pressure record.

### 3.2. Mussel Reactions to Noise

At this point, our aim is to verify whether mussels react to the noise caused by passing ships by modifying their shell opening-closing rhythms. For this purpose, we manually select, with the help of AIS data, analysis intervals in which the movement of particles shows noticeable increases due to the passage of ships. We chose the acceleration variable for this analysis because it is, a priori, the one that best detects the bivalve statocyst [22]. In particular, we show the results in the 1 kHz band, as this is the band that most clearly describes the level changes produced by ship passages, although the temporal evolution of the correlation results with the valvar aperture is similar in other bands.

**Figure 11:**
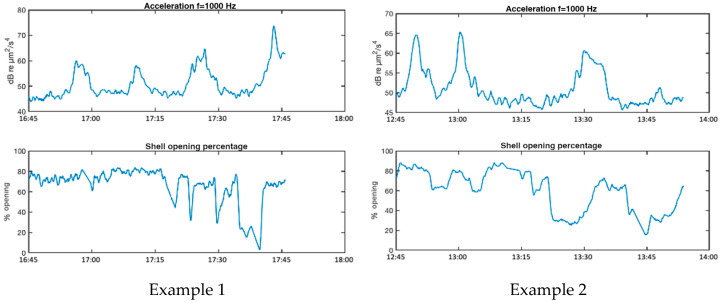

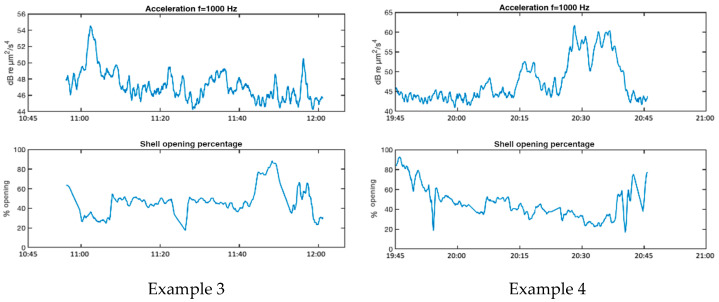
Examples of reactions of mussels to increased noise levels from passing ships.

Figure 11 shows four examples of reactions of mussels in AGU to noise increases produced by ship passages. Examination of the reactions depicted in examples 1 and 4, show that the noise peaks causing significant changes in shell opening rhythm have levels around 50–60 dB µm^2^/s^4^ of particle acceleration in the 1 kHz band, corresponding in our recordings to 75–85 dB µm^2^/s^4^ in the full band (63 Hz to 20 kHz).

Table 2 column 2 shows the linear dependence, LD, as defined in Equation (10), between the valve opening and the particle acceleration (column 1) and sound pressure (column 2) for the four examples. The LD value between valve opening and temperature (column 3) and between valve opening and raft motion due to waves, measured with an accelerometer (column 4), are also given to consider the possibility that the mussel reactions are due to variations in these conditions. The LD values shown retain the sign of the $R_{xy,coeff}$ coefficient obtained in (10). For all values shown in the table, *p* < 0.05 is satisfied.

The analysis of the LD values in Table 2 reveals significant negative correlation values between valve opening and particle acceleration, reflecting that the higher the particle acceleration level, the lower the valve opening, i.e., in these examples, the increased noise level causes partial valvar closure. Very similar results are obtained for the dependence between valvar aperture and sound pressure, revealing a close relationship between both acoustic variables in the frequency band of analysis. On the other hand, the LD values between valve opening and the other two environmental variables (water temperature and raft movement) show both positive and negative values, which seems to show that either there is no clear cause-effect relationship between these variables and the valve opening in the examples studied, or the different variables studied interact with other factors not analysed, generating opposite effects.

## 4. Discussion

The aim of this work is to analyse the reactions of mussels to underwater noise caused by ship traffic, establishing a new methodology of in situ monitoring based on more accessible technologies. Therefore, the experimental setup has been deployed at sea, rather than in a laboratory, as all previous studies on bivalves have been performed [9,10,11,12,13,14,15,16,17,18,19]. Experimenting at sea allows to accurately assess the impact of the particle motion generated by ship traffic noise, the main pollutant in mussel farming areas, which is not possible at a laboratory tank, but also to demonstrate the effectiveness of using HFNI valvometry as a monitoring system to detect stress in marine invertebrates caused by anthropogenic underwater noise.

An important aspect of the methodology followed in this project is the description of the reactions to the acoustic field in terms of particle motion variables rather than with sound pressure. However, suspending particle motion measuring instruments from the rafts makes it very difficult to obtain good quality measurements, because of the noise that would be introduced by the movement of the sensor itself under these conditions [25,32]. In this study, we performed sound pressure measurements with hydrophones because it was important to sample the acoustic field in the vicinity of the mussels, which are suspended from floating rafts in areas up to 45 m deep. Then, the hydrophone sound pressure measurements were transformed into particle movement variables using well-established equations.

The correct application of sound pressure to particle motion transformation requires the fulfilment of certain conditions. It must be estimated under the plane wave hypothesis [25], whose verification in this study is guaranteed by eliminating from the analysis the recordings corresponding to ship passages very close to the hydrophones (less than 24 m). The statistical description of the acoustic environment at the two experimental sites has been carried out in terms of the power spectral density of sound pressure and three particle motion variables (displacement, velocity, and acceleration). This description contributes to the general understanding of the behaviour of these variables, which are particularly important for invertebrates and most fish species, and are still little studied [22,32,33].

The difficulties inherent in an experiment in the sea are manifold. On the one hand, it is impossible to control environmental variables, which causes uncertainty as to what the real cause of a change in mussel behaviour is. In the examples analysed, we have been able to rule out the effect of raft movement and short-term variation in water temperature, but these are only two of the multiple stressors present in the habitat studied. However, in this regard, we can provide evidence that the maintenance of circadian rhythms of *Mytilus galloprovincialis* in a raft culture situation with respect to ‘almost permanent’ valvar opening has been previously described even though the set of abiotic/biological factors in the cultivation area fluctuates greatly (e.g., solar irradiance, primary production, temperature, salinity, etc.) [27].

Another difficulty of conducting experiments at sea has to do with the fact that the marine environment is very damaging to electronic equipment. During our experiment, the valvometry systems suffered numerous failures: cable breakage, detachment of sensors, failures in the data acquisition, storage, and transmission equipment. It has not been possible to solve many of them because of the difficulty of access to the rafts and the impossibility of handling the ropes on which the mussels studied are cultivated, due to the risk of them becoming detached. In fact, detachments have been significant in the two experimental study stations, associated with the effect of the presence of biofouling and hydrodynamic conditions, correlations that are still in the analysis phase.

For these reasons, the number of correctly monitored specimens is small, and we have only been able to obtain evidence of a cause-and-effect relationship between the noise of a passing ship and partial valvar closure in a few cases, some of which are shown in this contribution. From these examples, we have been able to estimate particle acceleration levels that cause partial valvar closure for specific individuals (75–85 dB re µm^2^/s^4^ in the band from 63 Hz to 20 kHz). Previous laboratory studies reported mussels reacting to ship noise levels above 114 dB in the band between 63 Hz and 4 kHz, with a lag of about 13 s after a sudden increase in sound level [15]. Nonetheless, we are aware that our results are not sufficient to draw valid conclusions for a cultivated biomass and communities, considering the high inter-individual variability of bivalve molluscs in general. The sample size to be tested needs to be significantly expanded to increase the statistical power and contemplate this inter-individual variability for a better understanding of the anthropogenic stressor thresholds that cause changes in the behaviour of cultured populations.

In future studies, to minimise failures in the valvometry systems, it would be advisable to look for an experimental site in protected waters and reduce the size of the colony in which the monitored specimens live. On the other hand, a site accessible from land would facilitate the tasks of monitoring the specimens and repairing the valvometry systems. In addition, an experimental deployment in shallower waters would enable the installation of equipment to measure particle movement, allowing a comparative study with particle movement estimated from sound pressure.

## Figures and Tables

**Table 1:** Location and depth of the water channel of the two rafts, identified by the first 3 letters of the nearest locality.

Raft ID (Nearest Locality)	Location	Channel Depth (m)
VIL (Vilagarcía)	42.6004 N, 8.8270 W	20
AGU (Aguiño)	42.5184 N, 8.9928 W	45

**Table 2:** Linear dependence between valvar aperture and particle acceleration, water temperature, and the modulus of raft acceleration.

	LD_open, accel_	LD_open, press_	LD_open, temp_	LD_open, raftaccel_
Example 1	−0.59	−0.59	0.55	−0.22
Example 2	−0.27	−0.26	0.06	0.56
Example 3	−0.50	−0.50	−0.47	0.26
Example 4	−0.64	−0.64	−0.52	−0.40

## Data Availability

Data available upon request to interested researchers.
